# Effect of short time precise dietary energy–protein in reproductive parameters of local crossbred dairy goats

**DOI:** 10.5455/javar.2023.j677

**Published:** 2023-06-30

**Authors:** Pradita Iustitia Sitaresmi, Mohammad Firdaus Hudaya, Seraphina Kumala, Herdis Herdis, Ahmad Sofyan, Sigit Bintara, Budi Prasetyo Widyobroto, Diah Tri Widayati

**Affiliations:** 1 Research Center for Animal Husbandry, National Research and Innovation Agency, Cibinong Science Center, Bogor, Indonesia; 2 Department of Animal Breeding and Reproduction, Faculty of Animal Science, Universitas Gadjah Mada, Yogyakarta, Indonesia; 3 Department of Animal Production, Faculty of Animal Science, Universitas Gadjah Mada, Yogyakarta, Indonesia

**Keywords:** Balancing nutrition, blood profile, energy, hormones, protein, Saanen crossbreeds

## Abstract

**Objective:**

This study aimed to establish and focus on the implications of precise energy and protein levels on reproductive performance. This study based on field facts showed that excessive feed intake, especially protein intake, to increase productivity will lead to some decreased reproductive efficiency through disruption of reproductive hormonal compound biosynthesis or increasing blood urea nitrogen (BUN), which has rarely been studied.

**Materials and Methods:**

Healthy and mature Saanen crossbred goats raised by traditional farmers (2–4 years old; ± 45 kg) were divided into three experimental groups [T0 = basal feed according to the feed provided by farmers, T1 = protein and energy balanced feed according to National Research Council (NRC) (±5%), T2 = feed >20% excess protein, and excess energy from NRC] (*n* = 75). The factorial method was used in the experimental design with a *post-hoc* least significant difference test.

**Results:**

The data showed a significant difference (*p < *0.05) in the biochemical or hormonal parameters between the control and the treatment groups. Also, T1 showed leads in any blood profile and reproductive hormone parameters such as total protein (79.6 ± 5.3 gm/dl), cholesterol (3.26 ± 0.47 mmol/l), progesterone (1.79 ± 2.21 ng/ml), and estrogen (47.85 ± 5.51 pg/ml), as well as the lowest levels of BUN (11.6 ± 1.59 mmol/l) and cortisol (25.07 ± 14.85 ng/ml) levels. T1 had the highest reproductive potential after treatment.

**Conclusion:**

The adverse effects of high and low protein consumption on reproductive hormones seem to be related to the blood profile and ovarian function, as hormone concentrations change significantly and lead to reproductive impairment. The data showed that balanced nutrient levels (5%/T1) resulted in excellent blood and hormone parameters.

## Introduction

The objective of feeding approaches is to enhance livestock performance by balancing nutritional needs based on the physiological status and feed ratios of the animals. The National Research Council (NRC) has well-established nutritional criteria for dairy goats [1]. This feeding approach had no practical or technological limitations [2]. The consequences of nutritional imbalance on the productive function of ruminants have been extensively studied and evaluated; however, few studies have been conducted on ruminants, especially regarding excessive feed rations. Previous research has found that excessive feeding, especially protein intake, to increase animal production leads to a decline in reproductive parameters [3]. Therefore, precise feeding is a strategy to adjust the monitoring of an animal’s nutritional process in order to provide optimum nutrition throughout time and among animals, which may indirectly improve animal health and well-being [4]. The most crucial stage was to illustrate the measurement of biological processes related to animal nutrition to identify the effects of precision feeding, which provides insufficient or excessive amounts of essential resources (e.g., energy, amino acids, vitamins, and trace minerals) to hinder reproductive performance.

Many recently published studies have stated that high-protein diets are provided to animals to boost productivity (elicit milk production and, more significantly, obtain high yields) [3]. Maximizing the production potential of dairy animals is now a conventional practice in dairy farming by using feed with a high protein content. However, according to several studies [3], increasing the proportion of crude protein (CP) in the diet decreases fertility. Recent research has demonstrated that ruminants given excessive protein (>15% over needs based on NRC [1]) have decreased reproductive performance, such as a high S/C rate, pregnancy ratio, kidding rate, low litter size, and accelerated luteolysis process. However, the mechanism by which this occurs remains unclear. In our previous study, we found that excess protein intake (>19% CP) in Saanen crossbreed goat feed potentially improves milk production but leads to impairment of reproduction by increasing blood urea nitrogen (BUN) and intruding reproductive hormones such as progesterone and estrogen to the limit of concentration, leading to decreased animal litter size from 2 to 3 to just one kid per partus time. It also shortens the estrous cycle, which is assumed to occur because the follicles that form tend to be small and limited [5]. This is because the high protein content in the diets of dairy animals decreases nitrogen (N) efficiency, increases BUN, and increases the excretion of N via urine and milk [6].

The increased BUN concentration may increase blood urea in the pre-ovulatory follicles and uterine fluid. Therefore, this condition leads to decreased fertility, such as embryo loss, due to altered circulating concentrations of reproductive steroid hormones [7]. However, the impact of excessive protein supplementation on dairy goats has not been extensively studied [8]. In addition to focusing on balancing nutritional intake, this research also focuses on the availability of feed supplies close to farms and the enhancement of a novel formulation customized to physiological and reproductive conditions [5]. This study aimed to compare two feeding regimens that mainly focused on energy and protein intake with control (the original feed ratio in smallholder farmers on non-lactating dairy goats based on reproductive performance in the anestrus phase and metabolic blood profile) to prove that severe nutritional deficiencies and excesses will disrupt metabolic processes and reduce reproductive parameters in small livestock, especially dairy goats raised in tropical climates. This research examines and specifies dietary nutrients in determining crossbred Saanen dairy animals’ physiological and reproductive status; it is also the continuation of previous research [9].

## Materials and Methods

### Ethical clearance

The Animal Care and Use Committee reviewed and approved all techniques used in this investigation by the Faculty of Veterinary University of Gadjah Mada, Number 00070/ECFKH/Eks./2021.

### Research location

During the dry season, the research was carried out at the Farm at Turi Yogyakarta and the Faculty of Animal Science at Universitas Gadjah Mada, Bulaksumur, Yogyakarta Special Region, in the southern part of Java, Indonesia, at 7 °599097675349111 S, E 110° 40277955490129, at an altitude of 580 m above sea level. During this time, the temperature ranged from 18°C to 28°C. The duration of the day at this location did not change considerably, ranging from 11 h 30 min to 12 h 45 min.

### Animals

**Figure 1.**
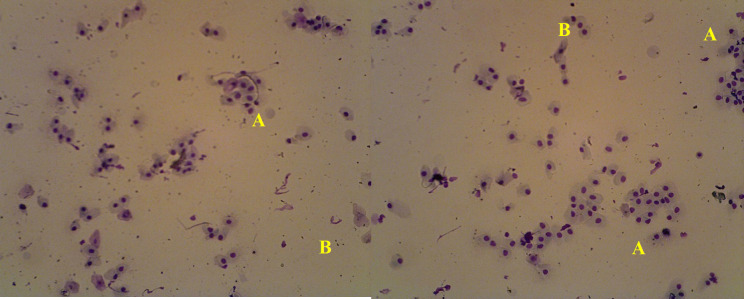
Photomicrographs of cytology vaginal from anestrus Saanen crossbreed goat. The drawn blood collected occurred on the anestrus phases which was proven by a swab vaginal smear using Giemsa staining 3% (Merck, Germany). Vaginal cytology in the anestrus phase predominantly consisting of nucleated epithelial cells (A) and leucocytes (B), under the influence of low levels of the hormone estrogen during the anestrous phase. Low levels of estrogen and relatively high blood levels of progesterone cause the outermost epithelial layer of the vagina to get enough nutrition from the blood so that it will form intact parabasal cells. These cells play an active role together with endometrial secretory cells in creating an acidic environment in the vaginal environment as a mechanism of protection from foreign substances or pathogens, and this is strengthened by the discovery of quite a number of leukocyte cells on the slide and strengthens this protection mechanism during the anestrus phase [10].

Dry, healthy Saanen crossbred goats (Saanen × Ettawah crossbreeds) (*n* = 75) were used in this study. The requirements of the goats were as follows: age ranged from 2 to 3 years old, had parturition 3 or 4 months earlier, and had finished lactating their kids. These goats weighed 40–45 kg and had normal (body condition scores: 2.5–3.5 out of 5 scales). All the goats were maintained in individual pens. The animals were in good health; blood was collected in the anestrus phase, as determined by vaginal smears and vaginal acidity (Fig. 1).

### Blood sampling, blood metabolites, and hormones analysis

Blood samples were obtained every morning at 6 a.m., thrice a week for 8 weeks, and the blood collection occurred in the anestrus phase during this research, which was confirmed using vaginal smear and vaginal acidity [10]. When the animals indicate heat or estrus, blood collection will be awaited until the goats do not show symptoms of estrus and data will be collected or grouped in 5 weeks in the anestrus stage. Blood from mature ewes was drawn from their jugular veins and placed in an Ethylene diamine tetra-acetic acid-treated tube. Isolated plasma was stored at a temperature of −20°C [11]. The metabolic profiles of the blood were analyzed (total protein, glucose (GLU), BUN, and cholesterol) using an ultraviolet spectrophotometer (Microlab 300, Italy). Hormone levels (including progesterone, estrogen, and cortisol) were measured using an Enzyme-linked immunosorbent assay kit (DRG Instruments GmbH, Germany). The kit’s horseradish peroxide conjugate was employed to detectably bind to the antibody coating [5]

### Animal treatment 

Feeding lasted 5 weeks in all animals, with pre-research lasting 3 weeks prior to using the basal ration (T0). The feed was supplied in the form of forage and concentrate, with feeding times of 08.00 (feeding concentrate) and 14.00 (feeding forage) (Table 1). The research had three treatments (T0, T1, and T2), each with 5 weeks of replication in the anestrus phase. Water is available at all times (*ad libitum*). As determined by the 2007 National Research Council, the dietary requirements of the goats are listed in Table 2.

The calculation of the adjustment between the basal feed (T0; *n* = 25) and the farmer’s feed and the goats’ daily nutritional requirements. Feed treatment 1 (T1; *n* = 25) was adjusted to meet the livestock nutritional demands in line with the 2007 NRC (±5%), which determined the adjustment between feeding and daily animal nutrient needs. Feed treatment 2 (T2; *n* = 25) surpassed the nutritional requirement of 20% based on the 2007 NRC, and the adjustment between feeding and the daily nutrient demands of livestock was determined, as shown in Tables 3 and 4. The basic demands for animal nutrition are adjusted by the weight of the Saanen crossbred utilized, which weighs ± 45 kg and was reared in a tropical climate.

### Statistical analysis

Three treatment groups were fed for 5 weeks on supplemental diets comprising different amounts of total digestible nutrients (TDN) and CP; significance was defined at *p < *0.05. Data were evaluated statistically by ANOVA and means, and SE was determined. The data are shown as the mean and SD. SPSS 26.0 for Mac (SPSS Inc., 2021) was used for all statistical analyses.

**Table 1.** Concentrated feed nutrient composition.

Feed ingredients	Nutritional content (%)				
	DM	CP	CF	Fiber	TDN
Commercial concentrated	88.94	12.39	6.49	15.91	68.00
*Calliandra* (*C. calothyrsus*)	30.31	15.14	0.68	7.22	60.37
Napier grass (*Pennisetum purpureum)*	23.28	9.92	0.40	7.67	62.30

DM, dry matter; TDN, Total Digestible Nutrient; CP, Crude Protein; CF, Crude Fat.

## Results

### Biochemical blood parameters in Saanen crossbred dairy goat

**Figure 2.**
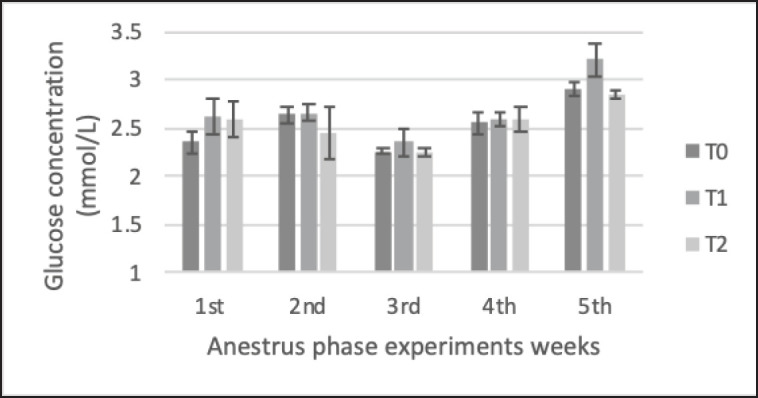
Changes GLU concentration Saanen crossbreed goat in each treatment during 5 weeks in the anestrus phase. All values are least square mean ± SD (*n = *75). GLU plasma concentration was analyzed by the Jaffe method, atomic absorption spectrophotometry (AAS) method using GOD-PAP methods. No significant difference (*p* > 0.05) in GLU data was found, and the GLU concentration was relatively stable during the experiment. Hemostatic ability stabilizes GLU levels as long as energy and protein needs do not exceed or fall below 35%, making the goat can maintain normal blood GLU levels. GLU ranges in normal concentrations (2.19–4.5 mmol/l). T1 and T2 had slightly higher GLU levels than T0 due to increased energy intake used in gluconeogenesis in control animals.

**Figure 3.**
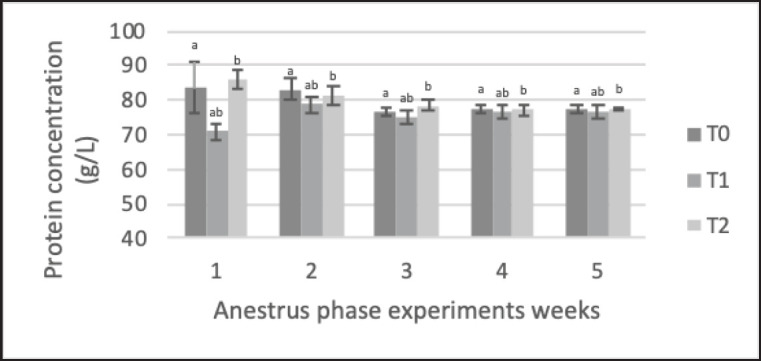
Changes protein (TP) concentration Saanen crossbreed goat in each treatment during 5 weeks in the anestrus phase. All values are least square mean ± SD (*n = *75). Total Protein plasma concentration was analyzed by the Jaffe method, AAS method using Bio-Rad Protein methods. TP profiles of each ration group varied significantly (*p* < 0.05). The high (T1 and T2) and low (T0) concentrations of TP in the blood depend on the amino acids absorbed through the intestinal wall, the linear protein profile was found to be consistent with the protein intake. ^a,b^Mean with different superscripts on the given graph showed a significant difference (*p* < 0.05).

**Figure 4.**
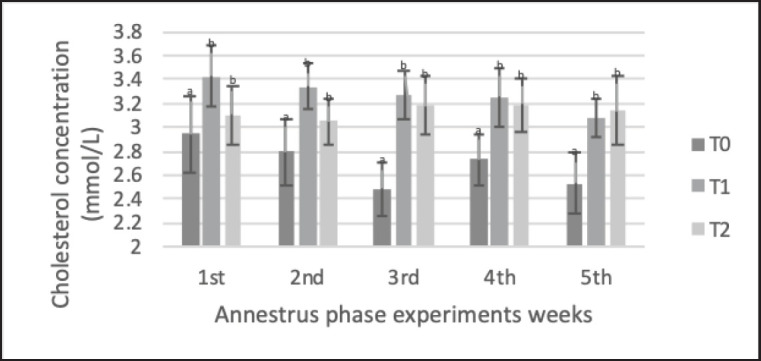
Changes cholesterol concentration Saanen crossbreed goat in each treatment during 5 weeks in the anestrus phase. All values are least square mean ± SD (*n = *75). Total cholesterol plasma concentration was analyzed by the Jaffe method, AAS method using CHOD-rad methods. Substantial differences (*p* < 0.05) in cholesterol concentrations in each ratio group. T1 had the highest cholesterol levels 3.26 ± 0.47 mmol/l. ^a,b^Mean with different superscripts on the given graph showed a significant difference (*p* < 0.05).

The results of biochemical parameters (Table 5) showed that precise feeding with adjustment for NRC [1] (with a range of ±5%) led to significant blood metabolic parameters. Average total protein and cholesterol levels were significantly higher in the treatment groups (T1 and T2) than in the control group (T0). Total protein (Fig. 3) in T1 and T2 (79.6 ± 5.3 and 80.1 ± 5.2 gm/dl, respectively) was significantly higher (*p < *0.05) than in the control group T0 (75.6 ± 8.2 gm/dl). cholesterol (Fig. 4) in T1 and T2 (3.26 ± 0.47 and 3.13 ± 0.5 mmol/l, respectively) were significantly higher (*p < *0.05) than in the control group T0 (2.69 ± 0.560 mmol/l). BUN (Fig. 4) in T1 (11.60 ± 1.59 mmol/l) was significantly lower (*p < *0.05) than in the control groups T0 and T2 (14.69 ± 2.6 and 13.54 ± 2.35 mmol/l); furthermore, the T0 BUN had a higher value than the reference values, which were <14 mmol/l. There was no significant difference in plasma GLU levels (Fig. 2) among the animals (*p* > 0.05).

**Table 2.** Nutritional needs of goat maintenance in the tropical environment [1].

Weight (kg)	Energy requirement				CP	
	TDN (gm)	DE (gm)	ME (Mcal)	NE (Mcal)	TP (gm)	DP (gm)
10	199	0.87	0.71	0.40	27	19
20	334	1.47	1.20	0.68	46	32
30	452	1.99	1.62	0.92	62	43
40	560	2.47	2.02	1.14	77	54
50	662	2.92	2.38	1.34	91	63
60	760	3.35	2.73	1.54	105	73
70	852	3.76	3.07	1.73	118	82
80	942	4.16	3.39	1.91	130	90
90	1030	4.54	3.70	2.09	142	99
100	1,114	4.91	4.01	2.26	153	107

TDN, Total Digestable Nutrient; DE, Digestible Energy; ME, Metabolized energy; NE, Net Energy; TP, Total Protein; DP, Digestible Protein.

**Table 3.** Feeding amount (gm BK/head/day).

Feed ingredients	T0		T1		T2	
	Conten (DM gm)	Content (% DM)	Conten (DM gm)	Conten (% DM)	Conten (DM gm)	Conten (% DM)
Commercial concentrate	444.70	67.47	711.52	71.96	800.46	67.56
Forages	214.36	32.53	288.17	28.03	384.35	32.44
*- Calliandra*	121.24	18.39	90.93	9.19	151.55	12.79
*- *Napier grass	93.12	14.13	186.24	18.84	232.80	19.65
Nutrient parameter						
*- *CP	82.69	12.54	87.34	8.83	101.14	8.53
*- *Crude fat	30.06	4.56	47.54	4.81	53.91	4.55
*- *Crude fiber	86.65	13.15	134.05	13.56	156.15	13.18
*- *TDN	433.60	65.79	654.76	66.22	780.84	65.90

DM, dry matter; TDN, Total Digestible Nutrient.

**Table 4.** Feed nutrient analysis correction.

	T0		T1		T2	
Nutrient	TDN	CP	TDN	CP	TDN	CP
Feed intake (gm)	433.60	82.69	654.76	87.34	780.84	101.13
Nutrient requirement (gm)	611.00	84.00	611.00	84.00	611.00	84.00
Correction (gm)	−177.39	−1.31	43.76	3.98	169.83	17.13
Correction (%)	−29,04	−1.56	+7.16	+3.98	+27.79	+20.40

TDN, Total Digestible Nutrient; CP, crude protein.

**Table 5.** Precise feed effect in blood profile parameters of the Saanen crossbreed.

Parameter	Group treatment			References values
	T0	T1	T2	
GLU (mmol/l)	2.54 ± 0.29	2.68 ± 0.41	2.54 ± 0.38	2.19–4.5 [13]
Protein (gm/l)	75.6 ± 8.20^a^	79.6 ± 5.3^ab^	80.1 ± 5.2^b^	60–82 [13]
Cholesterol (mmol/l)	2.69 ± 0.560^a^	3.26 ± 0.47^b^	3.13 ± 0.5^b^	1.99–3.37 [5]
BUN (mmol/l)	14.69 ± 2.6^b^	11.60 ± ^a^1.59	13.54 ± 2.35^b^	<14 [10]

^a,b^Mean with different superscripts on the same row and group showed a significant difference (*p *< 0.05).

### Reproductive hormone parameters in Saanen crossbred dairy goat in the anestrus phase

The results of the reproductive hormone parameters (Table 6) showed that precise feeding with adjustment in NRC [1] (T1) led to any reproductive hormone parameter. Average progesterone and estrogen levels were significantly higher in the treatment group than in the control group. The average cortisol level was significantly lower in the treatment group than in the control group. Estrogen levels in T1 and T2 (47.85 ± 5.51 and 46.98 ± 4.55 pg/ml, respectively) were significantly higher (*p < *0.05) than in the control group T0 (38.12 ± 4.70 pg/dl). Progesterone levels in T1 and T2 (1.79 ± 2.21 and 1.62 ± 1.98 ng/ml, respectively) were significantly higher (*p < *0.05) than in the control group T0 (0.06 ± 0.09 ng/dl). In this result, the study funded shows that progesterone and estrogen levels remain low in the anestrus phase. Furthermore, the T0 estrogen and progesterone had lower reference values. Cortisol levels in T1 (25.07 ± 14.85 ng/ml) were significantly lower (*p < *0.05) than those in the control groups T0 and T2 (46.98 ± 4.55 ng/ml and 47.85 ± 5.51 ng/l). Furthermore, T0 and T2 cortisol levels were higher than the reference values.

**Table 6.** Precise feed effect in hormonal parameters of the Saanen crossbred.

Parameter	Treatment group			References values
	T0	T1	T2	
Estrogen (pg/ml)	38.12 ± 4.70^a^	47.85 ± 5.51^b^	46.98 ± 4.55^b^	47–94 [5]
*R* square	0.75	0.81	0.68	
*p values*	0.25	0.19	0.32	
Progesterone (ng/ml)	0.06 ± 0.09^a^	1.79 ± 2.21^b^	1.62 ± 1.98^b^	1–5 [5]
*R* square	0.89	0.99	0.77	
*p values*	0.11	0.02	0.05	
Cortisol (ng/ml)	46.98 ± 4.55^b^	25.07 ± 14.85^a^	47.85 ± 5.51^b^	12–45 [5]
*R* square	0.681	0.85	0.81	
*p values*	0.32	0.15	0.19	

^a,b^Mean with different superscripts on the same row and group showed a significant difference (*p* < 0.05).

## Discussion

Prioritize the metabolic utilization of available energy in ruminants according to the relevance of each physiological condition as follows: 1) basal metabolism, 2) activity, 3) growth, 4) energy stores, 5) pregnancy, 6) lactation, 7) extra energy reserves, 8) estrous cycles and pregnancy, and 9) surplus and loss of energy reserves [12]. Because of the high metabolic demands of maintaining basal energy reserves and milk production, reproduction activity in dairy animals is restricted. Feeding limitations or excesses have been linked to reproduction disruption [13], which has been shown to reduce the future rate of kidding. The results of this study also showed that a well-balanced diet (nutrition intake and nutrition needed to meet equilibrium scale ±5%) is essential for reproductive efficiency and that this is proven in blood and hormonal parameters. Interestingly, in this study, excess protein intake (>20%) reduced fertility parameters at the biochemical and hormone levels, which could delay the first ovulation or estrus, decrease the conception rate, increase the number of open days, and shorten the estrous cycle in the future because protein is provided in excess in an animal, which is also similar to previous research [14,15].

### Effect of precise feeding on biochemical blood parameters in Saanen crossbred dairy goat

The blood biochemical profile is a primary parameter in determining feeding efficiency, which is subsequently used in assessing livestock health, physiological condition, possible pathological events, nutritional adequacy, and more [16]. This study determined blood profiles such as GLU, protein, cholesterol, and BUN in Saanen crossbreed goats during the anestrous phase after treatment in tropical conditions.

GLU has the function of regulation in some physiologic processes such as steroidogenesis, gonadotropin synthesis, and secretion. According to this study (Table 5; Fig. 2), the low and excess energy and protein treatments showed no significant differences between the GLU profiles of each ration group of Saanen crossbred goats; this data was similar to previous research [16]. This condition occurs because hemostatic ability stabilizes GLU levels as long as energy and protein needs do not exceed or fall below 35% and allows adult dairy animals to maintain normal blood GLU levels [17,18]. In this study, dairy goats’ GLU also ranged in normal concentrations from 2.19 to 4.5 mmol/l [13]. T1 and T2 seemed to have slightly higher GLU levels than T0 because of the increased energy intake used in gluconeogenesis at T0 [14]. The GLU profile can be affected by feed energy intake [18], even though this study found that feed feeding below or with excess <35% energy and protein resulted in no differences in blood GLU on Saanen crossbreed goats.

Proteins function as structural components in animal cells. Proteins and other components are required to construct most animal tissues and organs. Thus, proteins in animal diets are essential for tissue development and repair. In this study (Table 5; Fig. 3), the total protein profiles of Saanen crossbred goats in each ration group varied significantly. In agreement with the literature, which states that high and low concentrations of total protein in the blood depend on the amino acids absorbed through the intestinal wall, the linear protein profile was found to be consistent with the protein intake (*p < *0.05) of the provided ratio in this investigation, with T2 having the highest concentration [19] or, in another way, the increased feed CP would lead to a higher blood protein profile in ruminants, but some studies also declared that higher feed CP also enhanced BUN production, which will explain in this section, so it is important to determine and provide proper feed to increase efficiency in nutrient utilization. The total plasma protein can be successfully used to assess the modification and delivery of nutrients during goat production. In situations where dietary protein intake is insufficient (T0), blood total protein is used as an energy source (gluconeogenesis), resulting in low blood total protein levels but increased blood GLU levels (Table 5) [13,19].

The results of this study indicated substantial differences (*p < *0.05) in cholesterol concentrations in each ratio group (Table 5; Fig. 4). Based on nutritional demand, livestock-fed balanced rations (T0) had the lowest cholesterol levels. The lower cholesterol levels in the negative energy balance (NEB) condition in the T0 group of goats modify the endocrine system, which indirectly changes cholesterol metabolism [18]. Under these circumstances, the physiology of animals changes cholesterol metabolism by activating catabolism [20] and lowering the production of reproductive hormones (Figs. 6 and 7). Goat metabolism maintains T0 homeostasis by enhancing the reactivity and sensitivity of hepatic insulin, which increases the lipolysis of adipose tissue for energy. Plasma cholesterol is attributed to decreased cholesterol biosynthesis enzyme receptors, especially HMGCR, HMGCS1, and SREBF-2 receptor genes [21,22]. Under conditions of below-basic ratios (T0), body fat mobilization for energy production increased. Reduced lipogenesis and increased lipolysis reduce cholesterol levels in livestock below their nutritional demands [22]. Low nutritional absorption and high metabolic rates also lead to lower blood cholesterol levels in Egyptian goats. The higher forage ration content in the T2 group caused a slight decrease in the blood cholesterol content in Saanen crossbred goats.

**Figure 5.**
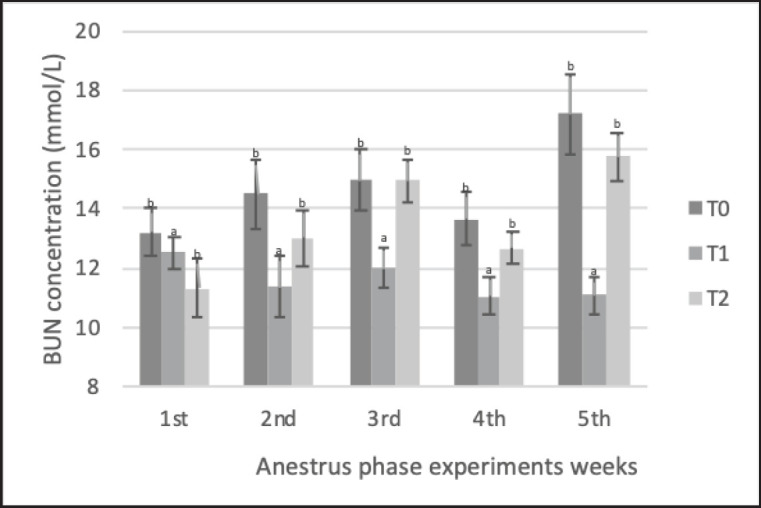
Changes ureum (BUN) concentration Saanen crossbreed goat in each treatment during 5 weeks in the anestrus phase. All values are least square mean ± SD (*n = *75). Total BUN plasma concentration was analyzed by the Jaffe method, AAS method using the urease-GLDH method. The data showed T1 significantly had the lowest BUN concentration during the treatment time. Avoiding excessive levels of BUN and plasma ammonia is feasible by adjusting the dietary energy and protein consumption rate, which could result in enhanced reproductive performance. ^a,b^Mean with different superscripts on the given graph showed a significant difference (*p* < 0.05).

**Figure 6.**
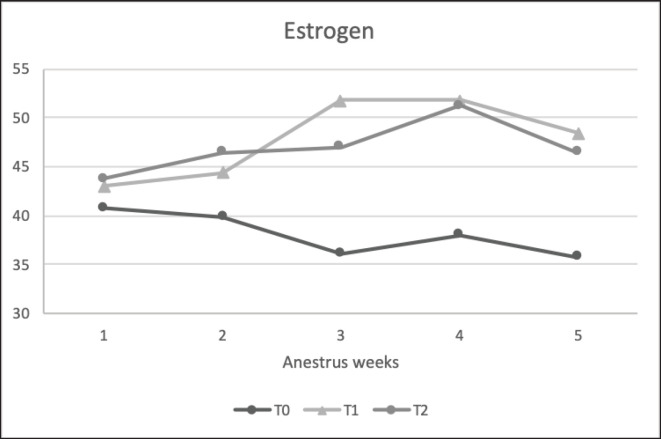
Estrogen (pg/ml) concentration Saanen crossbreed goat in each treatment during 5 weeks in anestrus stages*. *All values are least square mean ± SD (*n = *75). T1 and T2 were significantly higher (*p* < 0.05) than those of T0.

**Figure 7.**
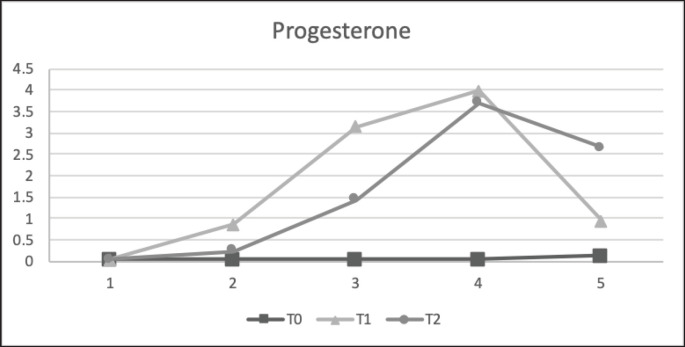
Progesterone (ng/ml) concentration in each treatment for 5 weeks in anestrus stages. All values are least square mean ± SD (*n = *75). T1 and T2 were significantly higher (*p* < 0.05) than those of T0.

In ruminants, the liver converts ammonia and nitrogen from protein deamination into urea, which is excreted via the kidneys and rumen [23]. In this study (Table 5; Fig. 5), BUN profiles in each ration group of Saanen crossbred goats differed significantly (*p < *0.05). Livestock fed according to nutritional needs (T1) had the lowest BUN levels. Untreated animals (T0) have nutritional contents below their demands, causing an NEB, hepatic fat mobilization, and the highest BUN concentration [24]. Under NEB conditions at T0, the catabolic mechanism of the kidneys increased BUN levels [25]. BUN levels indicate the breakdown of rumen protein and body tissues at T0, and normal BUN levels reflect a metabolic balance between T1 and T2. According to previous studies on Kacang goats, the increase in BUN levels was driven by the high protein content of the ration supplied, which impaired ovarian follicular growth by altering reproductive hormones [22]. Protein, a nitrogen source, increases rumen nitrogen breakdown, which, in turn, increases BUN levels (10). Avoiding excessive levels of BUN and plasma ammonia is feasible by adjusting dietary energy and protein consumption rates, which could result in enhanced reproductive performance [23].

A slightly higher result in T2 BUN was due to increased protein intake and protein breakdown in the goat body; this data was similar to previous research [21]. An increase of >3% of CP feed would increase nitrogen (N) utilization and result in an increase in BUN blood; a lower but balanced feed protein intake in T1 leads to efficient N utilization and a BUN level that remains at basal concentration [21]. Moreover, the T1 and T2 animals had normal urea levels (<14 mmol/l (11)); this indicates the efficiency of the feed protein provided. However, this could be due to the administration of the treatment in a short time, so if the treatment was carried out longer or using a higher concentration of protein feed, the accumulation of excess BUN would occur. This reinforces the fact that in this study the feeding at T2 has shown an increase in BUN and is an indication that the process of using blood N has begun to be inefficient. The inadequate consumption of calories, proteins, vitamins, and micro-and/or macrominerals has been linked to suboptimal reproductive function. Precise dietary intake can prevent excess BUN and excessively high plasma ammonia levels, which can lead to improved reproductive efficiency [26].

### Effect of precise feeding on reproductive hormone parameters in Saanen crossbred dairy goat in the anestrus phase

This study found a substantial link between ration treatment and progesterone levels (*p < *0.05), which matches goat studies [27]. Higher-nutrient (T1 and T2) goat feed raised blood progesterone levels [28], but undernourished goats (T0) had lower progesterone levels (15) (Fig. 7). Protein and energy supplementation boost pancreatic insulin output [29]. Insulin affects the ovarian tissue by boosting luteinizing hormone (LH) receptor production and pituitary activity through these receptors [30]. This condition maximizes follicular growth and creates a large corpus luteum (CL), which increases progesterone levels. The low level of progesterone makes it seem that this is an indication of the follicular phase or estrus phase, but based on the vaginal smear picture according to the previous picture (Fig. 1), which is in the anestrus phase in all animals used in this study, this is also reinforced by the fairly low level of estrogen obtained. This is because the smaller follicles in limited-fed ration goats (T0) produce less progesterone in their ovaries when they transform into the CL, but ovulation still occurs, considering the estrogen data, which is in the basal condition (Table 6).

This phenomenon has already been proven in previous studies, in which blood progesterone levels in SAPERA goats remained at basal levels for two successive cycles in all phases (estrus, metestrus, diestrus, and proestrus), caused by an imbalance in the nutrition of the feed given [31], even though the estrogen level remained stable. Other reasons for this phenomenon were the high BUN levels at T0 and T2, which had a detrimental influence in different ways. BUN creates an alkaline environment that increases PGF2α release and causes the CL to disappear, resulting in lower progesterone levels. A slightly lower progesterone concentration was observed in T2 than in T1, due to excess protein supplementation and increased BUN levels, affecting ovarian follicular and endocrine secretion. Excess protein consumption delays initial ovulation or estrus, reduces the conception rate, increases days open, shortens the estrus cycle, and lowers the overall conception rate, as indicated by low progesterone levels [13] (Fig. 1).

The results showed a significant difference between the control (T0) and treatment (T1 and T2) groups. Animals fed limited diets had decreased CL diameters owing to insufficient follicular development, as stated in the previous paragraph. Goats given limited rations or below their basal life nutrition need to experience an NEB, but the estrous cycle process still occurs. Only the length and number of waves produced in the cycle are affected, which differs from cattle, which showed that a lower nutrition-fed ration would directly affect the estrus cycle and could delay or inhibit the ovulation mechanism owing to differences in metabolism in cattle and goats [32]. Another study reinforced with ultrasound examination also found CL, which tends to be smaller and significantly inhibits or reduces the secretion of progesterone in the blood of livestock [29]. Our data revealed a decrease in progesterone concentration in too-high or too-low protein effects on endometrial activity, suggesting the potential participation of the prostaglandin–oxytocin system due to a more outstanding production of PGF2α in response to oxytocin; however, these findings need further study.

According to this study, nutritional treatments significantly affected estrogen concentration (Table 7; Fig. 6), which was also significantly correlated with cholesterol and BUN (Table 7). A shortage of ration nutrients, which affected the BUN and cholesterol profiles, was negatively associated with estrogen levels [33]. Similar to the cholesterol pattern, goats fed limited rations (T0) had the lowest estrogen levels. In animals fed restricted food for a considerable amount of time, the number of dominant follicles in the ovaries was minimal, resulting in low estrogen levels. Low cholesterol levels in this study also caused low estrogen levels because total cholesterol influences ovulatory steroidogenesis [34]. A previous study found that low blood GLU and cholesterol levels reduce LH pulses during follicular development, and LH functions as a factor in the formation of androgen hormones in granulosa cells to be converted into estrogen hormones. Hence, goats with limited rations also have low blood estrogen levels. The correlation test in this study (Table 7) demonstrated that the high concentrations of cholesterol and total protein could enhance the excretion of reproductive hormones, either steroids or protein hormones, and increase reproductive efficiency.

Goats fed a balanced diet have the highest levels of estrogen due to their high blood cholesterol levels [13]. Hence, precursors for estrogen synthesis are sufficient for ovarian steroidogenesis. Balanced rations help goats create optimum follicles and increase blood estrogen levels. This is also induced by increased GLUT-1 and GLUT-4 protein expression in granulosa cells and GLU absorption to maximize folliculogenesis and create more or larger dominant follicles. Thus, the quantity of estrogen released is greater, but the estrogen concentration is lower with excess positive energy (>20%). A negative feedback physiological system in the follicle suppresses estrogen production because it stimulates an increase in leptin, which negatively affects follicle estradiol secretion *in vivo* as a passive immunization mechanism to avoid hypersecretion of estradiol in the ovaries [13].

**Table 7.** Correlation between hormonal parameter tables.

Variable		Progesteron	Estrogen	Cortisol
Feed treatment	*p *correlation	0.343**	0.554**	0.025
	*p*	0.03	0.00	0.834
GLU	*p *correlation	0.050	0.050	−0.134
	*p*	0.672	0.670	0.250
Cholesterol	*p* correlation	0.138	0.297**	−0.351**
	*p*	0.237	0.010	0.002
BUN	*p* correlation	−0.171	−0.286*	0,389*
	*p*	0.143	0,013	0.01
Protein	*p *correlation	−0.090	0.013	−0.107
	*p*	0.441	0.911	0.361

*/** mean showed a significant (*p* < 0.05 / < 0.01) correlation in same columns and rows.

**Figure 8.**
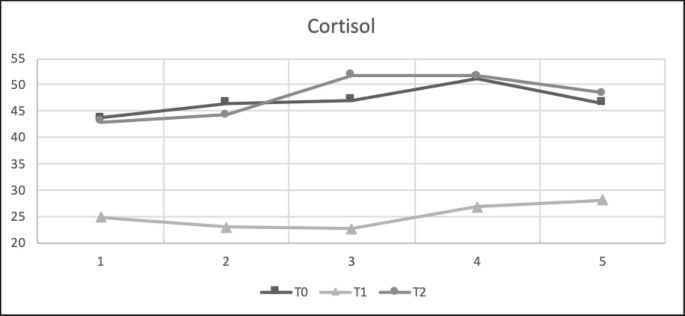
Cortisol (ng/ml) concentration in each treatment during 5 weeks in anestrus stages. All values are least square mean ± SD (*n = *75). T1 had significantly lower (*p* < 0.05) than the control T0 and T2. Testing with correlation and regression tests showed a close relationship between length time feed supplementation and all steroid hormone levels with a correlation >0.650.

Goat feed also affects cortisol hormones [22,32]. In goats given restricted feed (T0), blood cortisol levels increase as body fat and ration consumption decrease [35]. In this study (Table 6; Fig. 8), a high correlation was observed between blood cortisol levels and cholesterol and urea levels (*R*-values of −0.351 and 0.389, respectively). Cortisol negatively affects cholesterol, and imbalanced rations (T0 and T2) increase cortisol levels in goats. Cortisol is a hormone that is highly responsive to stress under conditions of cortisol imbalance in the blood, which increases and stimulates gluconeogenesis and lipolysis in the tissues [22]. This explanation follows the results of studies in which cortisol levels in the blood have a strong negative correlation (*R* = −0.351) with cholesterol levels, and cortisol levels have a negative connection with reproductive hormones such as estrogen (*R* = −0.246), since an increase in cortisol has a negative feedback effect on the hypothalamic-pituitary-adrenal.

The complicated influence of proteins on reproduction and insufficient protein intake reduce reproductive performance. The protein provided to a goat may affect its reproductive function. To reduce reliance on exogenous hormones in manufacturing environments, it is important to comprehend the manner in which dietary factors influence reproduction [35]. Tropical livestock are always energy-and protein-deficient. The weaknesses in this study that become suggestions for future research are the need for feeding for a longer time frame so that the effects of treatment will be obtained more comprehensively and may be able to answer some of the possible statements in this study.

## Conclusion

In this study, the adverse effects of high and low protein consumption on reproductive hormones seemed to be related to ovarian function since hormone concentrations changed dramatically. Although feed enhancement by increasing the quality and quantity of feed can increase livestock productivity, it is necessary to provide feed adjusted to the requirements of livestock to prevent pathological conditions either due to deficiency or excess of feed nutrients, especially in livestock reproductive parameters.

## List of Abbreviations

BUN, Blood urea nitrogen; CL, Corpus luteum; CP, Crude protein; dl, deciliter; e.g., exemple gratia; gm, gram; h, hours; kg, kilogram; l, Litter; LH, luteinizing hormone; min, minutes; mmol, millimole; N, Nitrogen; n, number; NEB, Negative energy balance; NRC, National Research Council; pg, picogram; TDN, total digestible nutrient; T0, control; T1, Treatment 1; T2, Treatment 2.
